# Do clinical trials affect anxiety, depression, and quality of life in the caregivers of patients with cancer?

**DOI:** 10.3389/fpsyt.2022.950787

**Published:** 2022-11-23

**Authors:** Xiaotong Guo, Lede Lin, Xiaohua Qiu, Meng Tian, Jiang Zhu

**Affiliations:** ^1^Department of Thoracic Oncology, West China Hospital, West China Medical Center, Sichuan University, Chengdu, China; ^2^Laboratory of Reconstructive Urology, Department of Urology, Institute of Urology, West China Hospital, Sichuan University, Chengdu, China; ^3^Department of Thoracic Oncology, West China Hospital, Sichuan University, Chengdu, China; ^4^Law School of Sichuan University, Chengdu, China; ^5^Department of Medical Oncology, Shangjin Nanfu Hospital, Chengdu, China

**Keywords:** anxiety, depression, quality of life, clinical trials, family caregiver

## Abstract

**Objective:**

To investigate the effect of clinical trials on anxiety, depression, and the quality of life experienced by the family caregivers (FCs) of cancer patients.

**Materials and methods:**

We screened the FCs of patients who were participating in clinical trials and FCs of patients who were not participating in clinical trials [group FCs-GCP (FG) and group FCs-non-GCP (FNG) at Cancer Center of West China Hospital]. We assessed the anxiety, depression, and quality of life of the FCs using the Hospital Anxiety and Depression Scale and SF-12. The demographic characteristics of FCs and patients were analyzed.

**Results:**

The prevalences of anxiety and depression showed no significant difference between FG and FNG (46.3 vs. 51.5%, *P* = 0.604; 36.6 vs. 51.5%, *P* = 0.131, respectively). Physical Component Scores (PCS) were 48.87 ± 7.67 for FG and 48.01 ± 8.12 for FNG (*P* = 0.618) while Mental Component Scores (MCS) were 48.92 ± 7.78 and 44.89 ± 11.42, respectively (*P* = 0.031). The anxiety of FCs was positively associated with patients’ advanced disease (HR 4.292 [1.409, 13.072], *P* = 0.010) and initial treatment (HR 3.105 [1.014, 9.515], *P* = 0.047). Depression was positively related to advanced disease (HR 3.347 [1.140, 9.832], *P* = 0.028), and negatively related to patients participating in clinical trials (HR 0.421 [0.180, 0.985], *P* = 0.046) and the education degree of FCs (HR 0.355 [0.149, 0.843], *P* = 0.019). MCS was positively associated with patients participating in clinical trials (β = 5.067, 95% CI [0.817, 9.317], *P* = 0.020) and negatively associated with advanced disease (β = −8.055, 95% CI [−19.804, 6.528], *P* = 0.002).

**Conclusion:**

The FCs of the cancer patients who participated in clinical trials showed a comparable worrying situation of anxiety and depression to the FCs of regular cancer patients. This indicates that more concern and attention should be given to this population, and further study on them is warranted.

## Introduction

Family caregivers (FCs) often play an essential role in providing care for patients with incurable cancer. Most FCs are patients’ spouses, parents, or adult children. They usually take on the responsibility of taking care of patients without preparation and instruction when patients are diagnosed. However, taking care of cancer patients can be complicated and tiring, including personal care, communication, management of medical care, helping with appointments, clinic visits, and providing emotional support (1). Such informal caregiving usually involves a great deal of time and some FCs even have to leave their jobs and stop working. Treatments also require money and financial support (2, 3). Under such caregiving burdens, FCs may experience psychological and physical distress, resulting in anxiety, depression, and poorer quality of life (QoL) (4).

The outcomes of randomized clinical trials are important for providing substantiated evidence for clinical decisions (5). To innovate and confirm more regimens of treatments for cancer patients, a number of clinical trials are processed in high-volume hospitals worldwide. Patients participating in clinical trials (GCP patients) have to follow the research design and process strictly with the assistance of clinical staff, which might save some time and money during treatment. But due to the study design, some patients do not know what treatments they receive, which might impact both patients and their FCs in terms of their psychological well-being. Regular patients who are not taking part in clinical trials and who are also treated in these hospitals might spend more time waiting for hospitalization. This long wait can delay their treatment and consequently affect their prognosis. Meanwhile, these delays can also have an adverse emotional impact on patients and their FCs.

Previous studies have explored anxiety, depression, and QoL in patients with cancer and their FCs (1, 4, 6–12, Supplementary Data Sheets 3, 4). To our knowledge, few studies to date have investigated the impact of patients participating in clinical trials on the anxiety, depression, and QoL of FCs. We investigated the effect of clinical trials on anxiety, depression, and QoL. Meanwhile, we explored the incidence of depression and anxiety and assessed QoL in FCs, trying to identify corresponding risk factors.

## Materials and methods

### Participants

In this study, all participants (FCs) were recruited when patients received treatments in the Department of Thoracic Oncology/Biotherapy, Cancer Center, West China Hospital, Chengdu, Sichuan, China, from October 2020 to June 2021. We enrolled the FCs of patients who were taking part in clinical trials and the FCs of patients who were not enrolled in the trials to explore the impact of participating in clinical trials on the anxiety, depression, and QoL experienced by FCs. All GCP patients received systematic anticancer treatments involved with lung cancer, esophagus cancer, mediastinal and pleural cancer, pancreatic cancer, and genitourinary cancer. The treatments mainly included chemotherapy, immunotherapy, and radiotherapy, which aimed at evaluating the benefits of new regimens. The study was approved by the Ethics Committee of West China Hospital of Sichuan University (approval number 20201020).

### Inclusion criteria and exclusion criteria

Patients’ FCs were eligible if they met the following criteria:

(1) patients were pathologically diagnosed with cancer and had underwent medical treatments for one or more cycles;
(2) FCs were at least 18 years of age;
(3) they could understand and fill in the questionnaires truly and correctly;
(4) they took care of patients at home and/or in the hospital;
(5) FCs voluntarily participated in this study and provided informed written consent.

Patients’ FCs were not suitable for this study for the following reasons:

(1) FCs had psychiatric conditions or intellectual difficulties.

### Sample size and groups

The sample size was calculated by the website tool: http://powerandsamplesize.com/. A previous study (6) conducted by Nipp et al. showed that the FCs of patients with incurable cancer reported a prevalence of anxiety, at a level of 42.2%. Based on this prevalence, we set a significant level at α = 0.05 and test efficacy at β = 0.1, with a group ratio of 1:1. We finally intended to enroll at least 100 patients in two groups. Based on whether patients participated in clinical trials or not, FCs were divided into group FCs-GCP (group FG, patients participating in clinical trials) and group FCs-non-GCP (group FNG, patients not participating in clinical trials).

### Measurement

#### Characteristics

The demographic characteristics of FCs were obtained, including gender, age, marriage, education degree, jobs, household income, relationship to patients, and the time they accompanied patients. We also collected patients’ information about their gender, age, insurance, type of cancer, disease stage, current treatment, and recent response evaluation based on RECIST 1.0 (Response Evaluation Criterion in Solid Tumor). We also collected information on whether patients received treatment locally or not.

#### Scales

We measured FCs’ psychological status and quality of life using Hospital Anxiety and Depression Scale (HADS, Supplementary Data Sheet 1) and SF-12 (A 12-Item Short-Form Health Survey, Supplementary Data Sheet 2) (13, 14).

#### Hospital anxiety and depression scale

Hospital anxiety and depression scale contains two 7-item subscales to assess anxiety and depression, and each item is rated on a four-point (0–3) to assess the severity of anxiety or depression symptoms in the past 4 weeks. Based on scores of the subscales, cases were divided into non-cases (0–7 points), doubtful cases (8–10 points), and definite cases (11–21 points). Previous studies had explored and confirmed the scales’ reliability and validity in a Chinese population (9, 15). In our research, we identified clinical anxiety and depression to be present when scores were over seven points (13).

#### SF-12

SF-12 includes 12 items involving eight concepts (physical functioning, social functioning, role limitations due to physical health problems, role limitations due to emotional problems, bodily pain, vitality, general health, and mental health) to evaluate participants’ quality of life (QoL). Based on the scores of 12 items and weighting algorithms, Physical Component Summary (PCS) and Mental Component Summary (MCS) were calculated to measure the FCs’ health status in physical and mental conditions (14). Higher scores suggest a better condition. The minimal clinically important difference was 4 points for PCS and 2 points for MCS (16).

### Data analysis

All analytical procedures were carried out by SPSS (IBM SPSS Statistics 25). The χ2 test was used to analyze category variables and the student *t*-test was used to analyze continuous variables. Depending on whether patients participated in trials or not, we identified psychological well-being and quality of life in two groups. We then used univariate and logistic regression analysis to explore the possible factors associated with clinical anxiety and depression, while univariate and multiple linear regression for QoL. Risk factors at *P* < 0.1 in the univariate regression analysis were included in the multiple regression analysis.

## Results

A total of 109 FCs were enrolled from October 2020 to June 2021, 41 in group FG and 68 in group FNG. Almost all FCs were married (94.5%) and were close to patients (63.3% were spouses of patients and 32.1% were first-degree relatives such as parents, children, and siblings). Detailed characteristics of patients and their caregivers are shown in Table 1.

**TABLE 1:** Characteristics of patients in or not in clinical trials and their FCs (GCP or Non-GCP).

	GCP (*n* = 41)	Non-GCP (*n* = 68)	*P-value*
**FCs’ gender**			
Male	13	16	0.349
Female	28	52	
Age	49.27 ± 12.03	46.49 ± 10.70	0.212
**FCs’ marriage**			1.000
Married	39	64	
Single	2	4	
**Relationship**			0.885
Spouses	27	42	
First-degree relatives	12	23	
Others	2	3	
**FCs’ education degree**			0.540
<High school	15	21	
≥High school	26	47	
**FCs’ Jobs**			0.813
Yes	25	43	
No	16	25	
**Household income (10,000 Yuan)**			0.072
<8	14	26	
≥8 and <15	19	18	
≥15	8	24	
**Accompany (days/week)**			0.689
0	1	4	
1–6	8	14	
7	32	50	
**Patients’ gender**			0.264
Male	28	53	
Female	13	15	
Patients’ age	57.68 ± 7.37	56.03 ± 10.56	0.339
**Patients’ insurance**			0.054
Yes	32	62	
No	9	6	
**Native patients**			0.980
Yes	20	33	
No	21	35	
**Disease stage**			0.107
I–III	5	17	
IV	36	51	
**Initial treatment**			0.437
Yes	6	14	
No	35	54	
**Recent response evaluation**			0.269
PR	12	11	
SD	11	18	
PD	8	23	
Not yet	10	16	
**Type of cancer**			0.015
NSCLC	20	36	
SCLC	6	13	
Esophagus cancer	3	9	
Mediastinal and pleural cancer	1	7	
Pancreatic cancer	8	3	
Genitourinary cancer	3	0	

GCP, patients in clinical trials; Non-GCP, patients not in clinical trials; first-degree relatives, FCs are patients’ parents or adult children or siblings; Accompany, time FCs accompany patients per week; PR, partial recession; SD, stable disease; PD, progressive disease; Not yet, patients had not assessed efficacy; NSCLC, non-small cell lung cancer; SCLC, small cell lung cancer.

**FIGURE 1:**
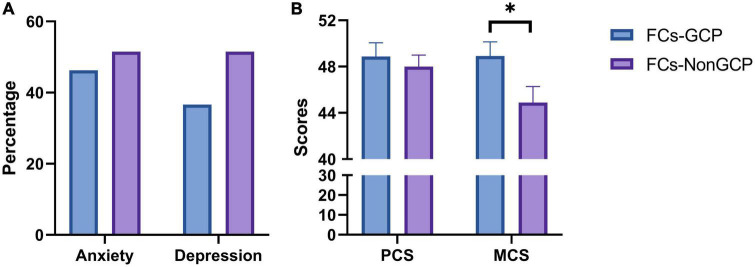
**(A)** Prevelance of anxiety and depression in FCs-GCP and FCs-Non-GCP. **(B)** Scores of SF-12 in FCs-GCP and FCs-Non-GCP. *P < 0.05.

The prevalence of anxiety and depression showed no significant difference between FG and FNG (46.3 vs. 51.5%, *P* = 0.604; 36.6 vs. 51.5%, *P* = 0.131, respectively, Figure 1). PCS were 48.87 ± 7.67 and 48.01 ± 8.12, respectively (*P* = 0.618, Figure 1), while MCS were 48.92 ± 7.78 and 44.89 ± 11.42, respectively (*P* = 0.031, Figure 1).

We used logistic regression to analyze risk factors of clinical anxiety and depression (Table 2) for all FCs. We found that FCs’ clinical anxiety was significantly associated with patients’ advanced disease (HR 4.292 [1.409, 13.072], *P* = 0.010) and initial treatment, which meant patients received only one cycle treatment (HR 3.105 [1.014, 9.515], *P* = 0.047). Higher clinical depression was positively related to patients’ advanced disease (HR 3.347 [1.140, 9.832], *P* = 0.028) and negatively related to patients participating in clinical trials (HR 0.421 [0.180, 0.985], *P* = 0.046) as well as the FCs’ education degree (HR 0.355 [0.149, 0.843], *P* = 0.019).

**TABLE 2:** Univariate and logistic regression analysis of anxiety and depression according to HADS.

	HADS-A			HADS-D		
		
Variable	Univariate	Logistic regression		Univariate	Logistic regression	
				
	*P-value*	*P-value*	HR (95% CI)	*P-value*	*P-value*	HR (95% CI)
Gender of FCs	0.254			0.895		
Age of FCs	0.757			0.420		
Marriage of FCs	0.658			1.000		
**Relationship**						
Spouses	0.890			0.030	0.110	
Immediate					0.403	
Others					0.105	
Education degree of FCs	0.635			0.025	0.019	0.355 (0.149, 0.843)
Jobs of FCs	0.190			0.749		
Household income	0.388			0.951		
Accompany (days)	0.299			0.523		
Clinical trials	0.604			0.131	0.046	0.421 (0.180, 0.985)
Gender of patients	0.621			0.417		
Age of patients	0.585			0.121	0.242	
Insurance	0.153			0.623		
Treated locally	0.504			0.301		
**Type of cancer**						
NSCLC	0.559			0.062	0.145	
SCLC					0.034	
Esophagus cancer					0.216	
Mediastinal and pleural cancer					0.839	
Pancreatic cancer					0.071	
Genitourinary cancer					0.071	
Disease stage	0.019	0.010	4.292 (1.409, 13.072)	0.050	0.028	3.347 (1.140, 9.832)
Initial treatment	0.126	0.047	3.105 (1.014, 9.515)	0.161		
Response evaluation	0.624			0.189		

HADS, Hospital Anxiety and Depression Scale; HADS-A, anxiety assessment according to HADS; HADS-D, depression assessment according to HADS; FCs, family caregivers; NSCLC, non-small cell lung cancer; SCLC, small cell lung cancer; HR, hazard ratio.

For FCs’ quality of life, we observed that higher MCS was positively associated with clinical trials (β = 5.067, 95% CI [0.817, 9.317], *P* = 0.020) and negatively associated with advanced disease (β = −8.055, 95% CI [−19.804, 6.528], *P* = 0.002).

## Discussion

### Clinical implications

Patients with cancer normally spend a great deal of time and money undergoing examination, treatment, and follow up. The majority of them suffer physical and mental symptoms during and after patients’ treatments (17). Thus, patients usually need both emotional and life support from their family members, most of whom are spouses, parents, adult children, or siblings. However, FCs are often exposed to various burdens, which lead to physical and psychological problems. The main psychological problems they encounter are anxiety and depression, while physical health problems include sleep disturbance and fatigue (1, 4, 7, 9, 10, 17, 18).

Randomized, clinical trials are commonly considered the highest level of evidence to support new regimens or drug entry into clinical practice. To ensure that the outcomes of clinical trials are ethical, reasonable, scientific, and convincing there are numerous guidelines and strict execution plans (5). For GCP patients, there is no need for them to worry about hospitalization and clinic visits, relieving part of their financial burdens and saving much time. They also can get more information and help about the disease from clinical staff while regular patients and their FCs complained that there was a lack of access to healthcare services and resources (17). However, the risks and benefits of these new drugs or new regimens are unclear and the prognosis is unpredictable. Uncertainty may also influence their emotional and physical conditions.

Recent studies have found that the prevalence of anxiety and depression in FCs were 42.2–48.9% and 16.4–25.5% (6, 7). In our study, the incidences were 46.3, 36.6% in group FG, 51.5 and 51.5% in group FNG, respectively, which were higher than previous reports. One of the reasons might be that most of the patients in this study (87/109, 79.8%) were diagnosed with stage IV cancers. Meanwhile, phase I/II clinical trials patients participated in accounted for about 41.5%. The benefits and risks of phase I/II clinical trials were not clear but they might bring anxiety and depression to the FCs of GCP patients. In addition, the scales for assessing anxiety and depression were not the same, which might induce a variance in scores. Compared with group FNG, group FG showed a lower incidence of anxiety and depression. Although there was no significant difference between the two groups, this demonstrated that participating in clinical trials might not increase FCs’ incidences of anxiety and depression.

We also found that the QoL of cancer patients’ FCs was worse than the general population in Sichuan. A study assessing QoL in Sichuan reported PCS and MCS in general people between the ages of 40 and 60 (PCS = 50.0 ± 6.5, MCS = 51.6 ± 6.8), which were higher than both group FG (PCS = 48.87 ± 7.67, MCS = 48.92 ± 7.78) and group FNG (PCS = 48.01 ± 8.12, MCS = 44.89 ± 11.42) (19). In this study, MCS in group FG was more than that in group FNG (48.92 vs. 48.01), which demonstrated clinical significance. Interestingly, group FG showed better mental conditions (MCS 48.92 ± 7.78 vs. 44.89 ± 11.42, *P* = 0.031) and was closer to the general population in Sichuan, which was considered to be of clinical significance (16). MCS evaluated participants’ QoL in mental health which includes anxiety, depression, and social function. Participating in clinical trials might relieve the burdens of medical services, reduce costs and enable patients to receive more attention from physicians, which would explain the higher MCS. As for physical conditions, group FG showed slightly higher scores than group FNG and we did not observe a significant difference between the two groups (48.87 ± 7.67 vs. 48.01 ± 8.12, *P* = 0.618). Thus, clinical trials might not affect and even do good for FCs, especially in terms of their mental health.

As the baseline in the study was slightly imbalanced, we used multiple linear and logistic regression to analyze risk factors associated with anxiety, depression, and MCS. Li has reported on the correlation of anxiety and depression between patients and FCs, which included the FCs’ gender, age, relationship to patients, education level, cancer types, and so on (9). In this study, the outcomes showed that increased clinical anxiety was related to advanced disease and initial treatment, while increased clinical depression was related to advanced disease. FCs of GCP patients and those with higher education levels were associated with a lower incidence of depression (Table 2). Better QoL in mental conditions was related to participating in clinical trials while poorer mental health was related to advanced disease (Table 3). Although the uncertainty of risks and benefits for patients participating in clinical trials might affect FCs’ anxiety, depression, and QoL, the outcomes of this study demonstrated that clinical trials did not seem to impact badly on depression and MCS. During the process of communicating with FCs, we also found that FCs with higher education degrees were well aware that the disease was nearly incurable and that treatments could extend a patients’ life and improve QoL, which might explain the lower incidence of depression. FCs with these risk factors should be recognized by physicians and nurses as early as possible. A detailed assessment and corresponding interventions or supports should be made to improve their conditions (20).

**TABLE 3:** Univariate and multiple linear regression analysis of MCS according to SF-12.

	MCS		
	
Variable	Univariate	Multivariate	
		
	*P-value*	*P-value*	β (95% CI)
Gender of FCs	0.119		
Age of FCs	0.908		
Marriage	0.723		
**Relationship**			
Spouses		0.871	0.781 (−8.159, 10.321)
Immediate		0.725	1.700 (−7.866, 11.266)
Others		Reference	1
Education degree		0.059	4.236 (−0.170, 8.642)
Jobs of FCs	0.556		
Household income	0.740		
Accompany (days)	0.750		
Clinical trials		0.020	5.067 (0.817, 9.317)
Gender of patients	0.120		
Age of patients	0.833		
Insurance	0.398		
Treated locally	0.420		
**Type of cancer**			
NSCLC		0.732	−2.205 (−14.960, 10.550)
SCLC		0.529	−4.150 (−17.194, 8.893)
Esophagus cancer		0.439	−5.305 (−18.852, 8.243)
Mediastinal and Pleural cancer		0.652	−3.420 (−18.411, 11.572)
Pancreatic cancer		0.319	−6.638 (−19.804, 6.528)
Genitourinary cancer		Reference	1
Disease stage		0.002	−8.055 (−13.048, −3.063)
Initial treatment	0.495		
Response evaluation criterion		0.088	1.665 (−0.251, 3.581)

SF-12, A 12-Item Short-Form Health Survey; MCS, Mental Component Summary; GCP, patients participating in clinical trials; FCs, family caregivers; NSCLC, non-small cell lung cancer; SCLC, small cell lung cancer.

It is important to also consider medical insurance. Most citizens in China have medical insurance that covers cover part of treatment costs. However, some cancer patients do not have insurance and some drugs have not been included in medical insurance, which means they suffer more of a financial burden. In this study the percentage of patients without medical insurance in group FG (22%) was more than that in group FNG (8.8%). This seemed to show that clinical trials might decrease the incidence of anxiety and depression, and improve the QoL for the FCs of patients without medical insurance.

### Study limitations

There were several limitations to our study. The study was carried out in one of the most famous hospitals in southwestern China, where patients commonly spend more time waiting for treatment compared with those treated in local hospitals. Additionally, we involved a few types of cancer with various prognoses. Thus, we plan to conduct further studies in some local hospitals later and involve more types of cancer. Moreover, the baseline characteristics seemed to not be comparable in our study, suggesting that selection bias might exist. We used multiple linear and logistic regression to analyze data, maximally reducing the influence of various factors on our outcomes. Finally, the emotional and psychological conditions of FCs changed with the disease course and patients’ response evaluation after treatments. Continuous assessment of FCs is needed in further investigations to explore related factors.

## Conclusion

Clinical trials might not increase the incidence of anxiety and depression, and worsen the QoL of FCs; they might even do them good, especially in terms of mental health. Higher clinical anxiety morbidity was related to advanced disease and initial treatment. Higher clinical depression morbidity was related to advanced disease while the lower incidence of depression was related to clinical trials and higher education levels. Better QoL in mental conditions was related to clinical trials while poorer mental health was related to advanced disease. More high-quality evidence is needed to certify conclusions in our study.

## Data availability statement

The original contributions presented in this study are included in the article/Supplementary material, further inquiries can be directed to the corresponding author.

## Ethics statement

The studies involving human participants were reviewed and approved by the Ethics Committee of West China Hospital of Sichuan University. The patients/participants provided their written informed consent to participate in this study. Written informed consent was obtained from the individual(s) for the publication of any potentially identifiable images or data included in this article.

## Author contributions

XG: conceptualization, data curation, formal analysis, funding acquisition, investigation, methodology, project administration, resources, software, validation, visualization, and writing – original draft and review and editing. LL: formal analysis, validation, and writing – review and editing. XQ and MT: data curation. JZ: conceptualization, validation, supervision, and writing – review and editing. All authors contributed to the article and approved the submitted version.
