# Supplementary material for: Do clinical trials affect anxiety, depression, and quality of life in the caregivers of patients with cancer?
Source: Front Psychiatry. 2022 Nov 23;13:950787. doi: 10.3389/fpsyt.2022.950787 (PMC9726870; doi:10.3389/fpsyt.2022.950787)
Supplement: Supplementary file 2 [file Data_Sheet_2.PDF]

Zung Self-Rating Anxiety Scale (SAS)

For each item below, please place a check mark (✓) in the column which best describes how often you felt or behaved this way during the past several days. Bring the completed form with you to the office for scoring and assessment during your office visit.

Place check mark (✓) in correct column.	A little of the time	Some of the time	Good part of the time	Most of the time
**1** I feel more nervous and anxious than usual.				
**2** I feel afraid for no reason at all.				
**3** I get upset easily or feel panicky.				
**4** I feel like I'm falling apart and going to pieces.				
**5** I feel that everything is all right and nothing bad will happen.				
**6** My arms and legs shake and tremble.				
**7** I am bothered by headaches neck and back pain.				
**8** I feel weak and get tired easily.				
**9** I feel calm and can sit still easily.				
**10** I can feel my heart beating fast.				
**11** I am bothered by dizzy spells.				
**12** I have fainting spells or feel like it.				
**13** I can breathe in and out easily.				
**14** I get feelings of numbness and tingling in my fingers & toes.				
**15** I am bothered by stomach aches or indigestion.				
**16** I have to empty my bladder often.				
**17** My hands are usually dry and warm.				
**18** My face gets hot and blushes.				
**19** I fall asleep easily and get a good night's rest.				
**20** I have nightmares.				

Source: William W.K. Zung. A rating instrument for anxiety disorders. Psychosomatics. 1971
