# Supplementary material for: Do clinical trials affect anxiety, depression, and quality of life in the caregivers of patients with cancer?
Source: Front Psychiatry. 2022 Nov 23;13:950787. doi: 10.3389/fpsyt.2022.950787 (PMC9726870; doi:10.3389/fpsyt.2022.950787)
Supplement: Supplementary file 3 [file Data_Sheet_3.PDF]

Patient Name:_______________________________________ Date:_____________________

Zung Self-Rating Depression Scale (SDS)

Instructions: For each item below, please place a check mark (√) in the column which best describes how often you felt or behaved this way during the past several days.

Place check mark (√) in correct column.	A little of the time	Some of the time	Good part of the time	Most of the time
1. I feel down-hearted and blue.				
2. Morning is when I feel the best.				
3. I have crying spells or feel like it.				
4. I have trouble sleeping at night.				
5. I eat as much as I used to.				
6. I still enjoy sex.				
7. I notice that I am losing weight.				
8. I have trouble with constipation.				
9. My heart beats faster than usual.				
10. I get tired for no reason.				
11. My mind is as clear as it used to be.				
12. I find it easy to do the things I used to.				
13. I am restless and can't keep still.				
14. I feel hopeful about the future.				
15. I am more irritable than usual.				
16. I find it easy to make decisions.				
17. I feel that I am useful and needed.				
18. My life is pretty full.				
19. I feel that others would be better off if I were dead.				
20. I still enjoy the things I used to do.				

Scoring the Zung Self-Rating Depression Scale

In scoring the SDS, a value of 1, 2, 3 and 4 is assigned to a response depending upon whether the item is worded positively or negatively.

For items 1, 3, 4, 7, 8, 9, 10, 13, 15, 19 the scoring is:

- A little of the time = 1
- Some of the time = 2
- Good part of the time = 3
- Most of the time = 4

Items 2, 5, 6, 11, 12, 14, 16, 17, 18, 20 are reverse scored as follows:

- Most of the time = 1
- Good part of the time = 2
- Some of the time = 3
- A little of the time = 4

The SDS index is derived by dividing the sum of the values (raw scores) obtained on the 20 items by the maximum possible score of 80, and expressed as a decimal point. The table below converts raw scores into SDS index scores.

A Table for the Conversion of Self-Rated Raw Scores to the SDS Index

Raw Score	SDS Index	Raw Score	SDS Index	Raw Score	SDS Index
20	0.25	40	0.50	60	0.75
21	0.26	41	0.51	61	0.76
22	0.28	42	0.53	62	0.78
23	0.29	43	0.54	63	0.79
24	0.30	44	0.55	64	0.80
25	0.31	45	0.56	65	0.81
26	0.33	46	0.58	66	0.83
27	0.34	47	0.59	67	0.84
28	0.35	48	0.60	68	0.85
29	0.36	49	0.61	69	0.86
30	0.38	50	0.63	70	0.88
31	0.39	51	0.64	71	0.89
32	0.40	52	0.65	72	0.90
33	0.41	53	0.66	73	0.91
34	0.43	54	0.68	74	0.92
35	0.44	55	0.69	75	0.94
36	0.45	56	0.70	76	0.95
37	0.46	57	0.71	77	0.96
38	0.48	58	0.73	78	0.98
39	0.49	59	0.74	79	0.99
				80	1.00

Citation: Zung WWK: A self-rating scale for depression. Archives of General Psychiatry 12:63-70,1965
